# A systematic review for improper application of nasal spray in allergic rhinitis: A proposed role of community pharmacist for patient education and counseling in practical setting

**DOI:** 10.5415/apallergy.0000000000000173

**Published:** 2025-01-13

**Authors:** Anmar Al-Taie

**Affiliations:** ^1^Clinical Pharmacy Department, Faculty of Pharmacy, Istinye University, Istanbul, Türkiye

**Keywords:** Allergic rhinitis, community pharmacist, nasal spray, proper technique, topical preparations

## Abstract

The application of nasal spray is encountered with technique errors, which can lead to decreased therapeutic response and treatment failure. Community pharmacists can play a pivotal role in providing appropriate knowledge and counseling services for the proper and effective use of these topical drugs. The aim of this systematic review was to assess the most important aspects of application technique errors and the impact of community pharmacist-led interventions on the provision of patient education and counseling regarding the application of these topical preparations in clinical practice. Preferred reporting items for systematic review and meta-analysis (PRISMA) criteria were used to set up a systematic search through different databases, including Scopus, Web of Science, and PubMed. A total of 10 articles were included in this study. Nearly three-quarters of the publications discussed improper technique, poor knowledge about installation technique, and poor adherence. Only 2 studies discussed pharmacist intervention, which comprised individualized brief education and training on the correct use of the nasal spray. The study highlights that there are many different aspects of application errors encountered by patients while using nasal spray. The study also highlights that there is a dearth of involvement of community pharmacists and pharmacist-led interventions for proper technique and application of these topical preparations.

## 1. Introduction

In clinical settings, health literacy is the level of an individual’s ability to receive and understand the basic health-related information and services necessary to make good health decisions [1]. The difficulty in understanding and effectively responding to the diverse health information associated with various medical conditions and treatment plans is known as low or limited health literacy. Poor literacy levels can be a major cause of inappropriate drug use, poor medication adherence, and ineffective drug effects, leading to a high incidence of adverse drug reactions and other drug -related problems, along with frequent admissions and hospitalizations [2]. The World Health Organization (WHO) reported that irrational medicine usage is a global health issue, with more than half of all prescription medicines being taken incorrectly [3]. This means that patients might not get the required therapeutic outcomes related to misunderstandings of proper drug administration provided by their healthcare professionals, including the pharmacists. Patients usually prefer to follow pharmacist’s instructions if they realize the real benefits of taking their medicines regularly and that stopping medication abruptly can cause an adverse effect. Most importantly, providing the most comprehensive information will improve patient health by empowering them to take more control over their health [4, 5].

Allergic rhinitis (AR) is an inflammatory allergic disorder of the nasal mucosa characterized by the production of an immunoglobulin E (IgE)-mediated immune response toward allergens [6]. It frequently coexists with other allergic disorders, such as asthma, resulting in decreased quality of life [7]. AR is a global concern since its prevalence has increased over time, affecting 400 million individuals globally [8]. It is estimated to affect 25% and 40% of children and adults worldwide, respectively. Approximately 80% of AR symptoms appear before the age of 20 years [9], peaking between the ages of 20 and 40 before gradually declining [10]. The prevalence of AR varies by gender, males have a higher incidence of AR in childhood, whereas females have a higher prevalence in adolescence [11, 12]. On the other hand, it has been claimed that AR is more widespread in urban settings than in rural areas [13]. Several previous studies comparing AR prevalence in urban settings versus rural areas and found that the prevalence of AR has grown over time due to a variety of risk factors, including global urbanization [13, 14]. This is mostly caused by elevated levels of contaminants, which might aggravate pollen-sensitive AR [15]. Furthermore, climate change has been linked to longer pollen seasons in Europe over the past 3 decades, as well as increased seasonal allergies [16].

Nasal sprays are among the topical self-medications that are highly effective in treating and improving the quality of life for patients with AR. They act to deliver drugs to the nasal cavity on a local level, and most of the available formulations are effective with a single daily dose, reflecting efficacy, convenience, and safety with few side effects [17, 18]. The administration of these topical preparations consists of multiple action steps. Supplementary website, http://links.lww.com/PA9/A50 illustrates the required steps of proper nasal spray technique [19]. The ideal administration of nasal spray is spraying with a contralateral spray technique, pointing the nozzle away from the septum. This is because spraying toward the septum causes more nosebleeds than the suggested approach [20]. In addition, it is critical to maintain a neutral head position and inhale deeply when spraying. The most effective way to distribute the drug is with this strategy [21, 22]. Nasal sprays are self-medication medications that can be harmful if patients are not properly informed about how to use them.

In clinical practice, the application of nasal sprays is encountered with application technique errors, and many patients may find it difficult to administer them properly [23, 24]. Improper nasal spray application is determined when there is a lack of the recommended essential steps for application, despite the complete instructions for administration described in the patient information leaflet [19]. There is nonadherence to self-administered nasal medicines due to inadequate instructions, poor patient-provider interactions, and patients’ disagreement with the need for therapy [25]. Even after receiving instructions, many users continue to use nasal spray inappropriately [26]. A previous study found that most of the patients (94%) did not take their nasal spray as described in the patient information leaflets [27]. Therefore, proper application is considered highly important to avoid medication nonadherence, poor disease control, decreased therapeutic response, increased costs, treatment failure, and lower patient satisfaction and quality of life [28].

Among the most accessible healthcare providers are community pharmacists, who can play an important role in providing appropriate education, knowledge-based, and counseling services regarding the rational, safe, proper, and effective use of these topical drugs. This is because a large number of pharmacies are available in suitable and accessible locations, the long daily opening hours, and the fact that pharmacists can be reached without an appointment [29]. In contrast with the numerous reports evaluating the pivotal role of community pharmacists in the proper administration technique of other topical dosage forms, such as inhaler devices [30–32], the amount of literature on the administration of these topical preparations is dearth. In this vein, the aim of this systematic review was to assess the most important aspects of application technique errors regarding nasal spray application, along with the crucial role and impact of community pharmacist-led interventions toward the provision of patient education and counseling regarding the proper application of these topical preparations in a practical setting.

## 2. Methods

### 2.1. Study resources

A literature search was conducted as part of the search strategy for this study review in databases such as Scopus, Web of Science (WS), PubMed, International Scientific Indexing, and search engines like Google Scholar. The search strategy in the references and database was conducted between the years 1990 and 2023. The study applied the terms of medical topic headings and combinations of the keywords selected accordingly to identify articles with information relevant to the research: “allergy,” “allergic rhinitis,” “community pharmacy,” “community pharmacist,” “nasal inhaler,” “nasal spray,” “nasal spray technique,” “improper nasal spray technique,” “nasal spray non-adherence,” “pharmacist intervention,” “pharmacist education.”

### 2.2. Research eligibility and data collection

The titles and abstracts of eligible studies identified through the keywords collected from the search sources and databases were included in the research criteria in accordance with Preferred reporting items for systematic review and meta-analysis (PRISMA) standards and the preferred reporting items for systematic reviews. The potentially relevant articles were retrieved for a thorough full-text assessment as inclusion criteria using the following objectives: (1) study subject: nasal spray; (2) study type: retrospective, prospective cohort, case-control, clinical, and randomized controlled trials; (3) language: English. The following objectives were removed from the study and research process as exclusion criteria: (1) peer-reviewed publications in a language other than English; (2) publication type, including letters, comments, editorials, case reports, review articles, and animal studies; and (3) no use of nasal spray or medications.

To minimize bias assessment, an appraisal method, the Critical Appraisal Skills Programme checklist for systematic reviews, was used to assess the validity of the study, its results, and its overall quality. The research carefully followed the selection criteria to screen, observe, and extract publication data from the included article. An independent assistant with expertise in medical research projects also reviewed the quality and reliability of the information provided to rule out any duplicates. Based on the following data, the findings were narratively summarized for each extracted publication: author, year of publication, type of study, sample size, study setting, study parameters/intervention, study findings, and study main outcomes.

## 3. Results

### 3.1. Study selection and characteristics

**Figure 1.**
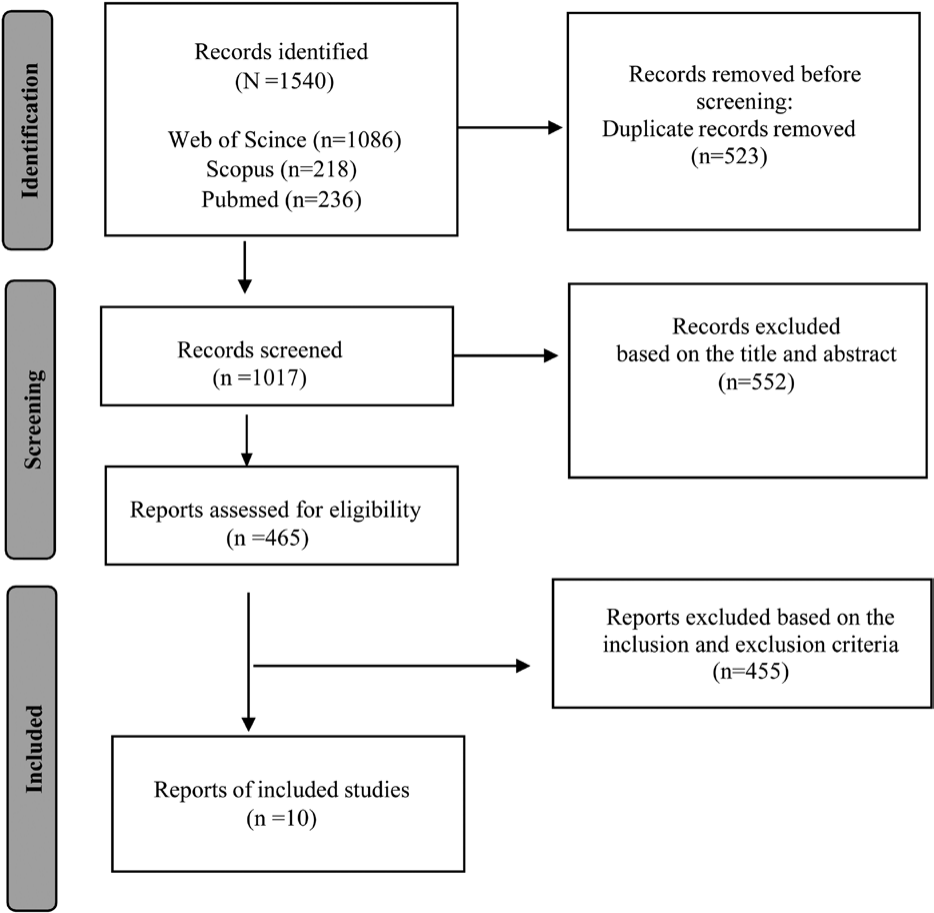
Flowchart for searching for studies in a systematic review.

We identified a total of 1540 studies. Articles were retrieved from the following databases: Web of Science (n = 1086), Scopus (n = 218), and PubMed (n = 236). However, a total of 465 articles were assessed for study eligibility, and 455 were excluded based on the study inclusion and exclusion criteria. As a result, a total of 10 relevant articles were included in the final review and analysis, as shown in Figure 1.

A total of 10 relevant articles were included in the final analysis, as shown in Tables 1 and 2. Nearly three-quarters of the publications (n = 7, 70%) were designed as cross-sectional, observational studies [23, 34–38, 40]. Only 1 study was conducted as a prospective pre- and post-interventional study [24]. Six out of 10 articles were carried out at the community pharmacy (60%) [34–38, 40], followed by 3 publications at the university hospitals [23, 24, 33]. However, the study by Canonica et al. [39] enrolled participants via computer-assisted telephone interviews. Most of the publications (70%) were conducted among patients [23, 24, 33–37]. Nevertheless, 3 publications were conducted among community pharmacists [38–40]. On the other hand, 2 studies involved pharmacist intervention, which comprised individualized brief education and training on the correct use of nasal spray with the help of a pictorial leaflet and the management of AR [24, 40]. The number of participants enrolled in the included studies ranged from 63 to 376, with the largest sample size enrolled in the study of Russo et al. [38], which was conducted among community pharmacists. Nearly three-quarters of the publications discussed improper nasal spray use and AR treatment (n = 7, 70%) [23, 33–38], as shown in Table 1. Meanwhile, 3 publications discussed the intervention by community pharmacists for proper application technique of nasal spray (n = 3, 30%) [24, 39, 40], as shown in Table 2.

**Table 1.** Summary of findings regarding improper nasal spray use*

Author	Year/country	Study design	Total number of participants	Study setting	Study parameters/intervention	Study findings	Study outcomes
Loh et al [33].	2004/Singapore	Comparative prospective study	63 (patients)	University Hospital	Compliance after 30 days of therapy: • Direct questioning • Measuring the weight of medication consumed Treatment efficacy was used to determine nasal symptom scores	77.8% reported a forgetfulness of using medication for a few times. A significant improvement in mean total symptom score before and after treatment (*P* < 0.001)	It is crucial to determine the true compliance when assessing patients who do not respond to intranasal steroid medication
Lourenço et al [34].	2014/Portugal	Cross-sectional study	224 (patients)	Community pharmacy	Control of Allergic Rhinitis and Asthma Test (CARAT) questionnaire	Median CARAT score = 19 87% of participants had a score < 25, indicating noncontrolled disease	Pharmacists can assist in the early detection of uncontrolled AR, which is a crucial first step in educating patients about their condition and enhancing AR outcomes.
Tan et al [35].	2017/Australia	Cross-sectional observational study	224 (patients)	Community pharmacy	Research-administered questionnaire	69.9% self-managed with over-the-counter medications Medication selection was mainly based on pharmacy customers’ perceptions of medication effectiveness (47.6%).	Medication purchasing patterns suggest that suboptimal therapeutic decisions made by participants in the community pharmacy
Tan et al [36].	2018/Australia	Cross-sectional observational study	296 (patients)	Community pharmacy	Pattern of AR symptoms QoL Medication(s) selected	69.3% self-selected medication(s) and were 4 times more likely to experience moderate-severe wheeze and 0.4 times less likely to experience an impact on QoL	An opportunity for pharmacists to interact with patients and promote conversation regarding the management of AR
Tan et al [37].	2018/Australia	Cross-sectional observational study	296 (patients)	Community pharmacy	Pattern of AR symptoms QoL Medication(s) selected	70% of participants self-selected their medications. 16.5% of participants selected optimal medications	Pharmacists need to take a proactive and evidence-based role in the management of AR
Almutairi et al [23].	2020/Suadi Arabia	Cross-sectional study	375 (patients)	Hospital-Medical Center	Research-administered questionnaire	73.3% did not receive appropriate advice on how to use intranasal corticosteroids. 53.9% reported being aware of a special technique for how to use a nasal spray	Suboptimal patients’ knowledge about, adherence to, and perceptions of intranasal corticosteroid use
Russo et al [38].	2023/Italy	Cross-sectional study	376 (pharmacists)	Community pharmacy	Research-administered questionnaire	44.4% of patients used higher than recommended dosage. Longer duration than 5 days in up to 31.9% of the cases. AR was the most common disease affecting patients seeking sympathomimetic amines.	Long-term usage of sympathomimetic amines in patients with rhinology diseases is a serious issue that needs more attention in terms of social education and surveillance.

Control of Allergic Rhinitis and Asthma Test

AR, allergic rhinitis; QoL, quality of life

* Articles are ordered according to the publication year.

**Table 2.** Summary of findings regarding healthcare professionals’ intervention for proper nasal spray technique*

Author	Year/country	Study design	Total number of participants	Study setting	Study parameters/intervention	Study findings	Study outcomes
Canonica et al [39].	2015/Italy	Descriptive survey-based study	100 (pharmacists)	computer-assisted telephone interviews	Diagnosis and treatment, degree of everyday limitation from AR Satisfaction with treatment.	Pharmacists considered eye tearing (54%) 87% of pharmacists were unaware of the AR and its impact on Asthma (ARIA) guidelines. Pharmacists recommended an antihistamine for 56% and a nasal decongestant for 21% of clients.	Pharmacists’ involvement in programs for allergy awareness and management
Arsoy et al [40].	2018/Cyprus	Cross-sectional survey	70 (pharmacists)	Community pharmacy	Pharmacists provided a brief education on management of AR and nasal spray technique for patients after a 6-week period. QoL assessment	Significant improvement regarding nasal congestion and QoL after pharmacists-led educational intervention (*P* < 0.05)	An educational intervention of the pharmacists can enhance the symptom management and improve the QoL in patients with AR
Kc et al [24].	2020/Nepal	Prospective pre- and post-interventional study	81 (patients)	Hospital	Pharmacist intervention comprised individualized education and training on the correct use of nasal spray with the help of a pictorial leaflet in the local language over 10 min. using a standardized WHO nasal spray checklist	↑ 9.84 ± 1.699 after intervention 4.31 ± 1.625 before intervention ↑ correct use by 50.275 of the subjects after the intervention Effective intervention on nasal spray use technique (*P* = 0.0001).	A pharmacist’s involvement significantly improved the ineffective nasal spray application technique.

AR, allergic rhinitis; QoL: quality of life.

* Articles are ordered according to the publication year.

### 3.2. Improper application technique and related errors

The lack of patient education and awareness of the benefits of topical nasal spray has been cited as another key barrier to medication adherence. Poor instructions, poor patient-provider interactions, and patients’ disagreement with the need for therapy all contributed to nonadherence to self-administered drugs. Even after receiving instructions, many patients use the sprays inappropriately [23, 24, 33]. A study by Kc et al. [24] found that most participants found the nasal spray application technique inefficient due to different factors. These included insufficient instructions provided at the time of dispensing, patients forgetting how to apply it, not understanding the significance of each step, and a lack of sufficient knowledge of how to clean and handle the spray.

Similarly, Almutairi et al. [23], in a cross-sectional study conducted in Saudi Arabia, reported that 73.3% of patients did not receive appropriate advice on how to use intranasal corticosteroids. Furthermore, 2 studies by Tan et al. [35, 37] found that around three-quarters of the patients reported self-selected medications, and 16.5% of them selected optimal medications. Russo et al. [38] in a study conducted in Italy among patients attending the community pharmacy using a research-administered questionnaire found that 44.4% of patients were using a higher than recommended dosage applied for sympathomimetic amines.

In a comparative prospective study by Loh et al. [33], using a clinical survey to evaluate the compliance in the treatment of AR among patients using nasal steroids, it was found that 77.8% of patients forgot to use nasal steroid medication nearly 1–5 times during the treatment period. Another cross-sectional study by Lourenço et al. [34] carried out in a community pharmacy found that 87% of participants had uncontrolled disease through the application of the Control of Allergic Rhinitis and Asthma Test. The study by Tan et al. [36] found that 69.3% of the participants reported self-selected medication(s) and were 4 times more likely to experience moderate-severe wheeze and 0.4 times lower quality of life (QoL).

### 3.3. Intervention of healthcare professionals in patient knowledge and education

In descriptive research conducted in Italy by Canonica et al. [39] using computer-assisted telephone interviews with community pharmacists, it was found that 56% of the clients who sought advice were given an antihistamine, and 21% were given a nasal decongestant. Patients rated AR 5.7 out of 10 for its impact on daily life. In addition, 55% stated they had used many therapies, and 43% stated their current course of treatment left them weakly or unsatisfied. The study’s findings suggested that allergy education programs need to be better targeted to pharmacists, and communication regarding symptom control must be improved with patients.

In a study by Kc et al. [24], pharmacist intervention included individualized teaching and training on the proper use of nasal spray with the use of a pictorial leaflet over the course of 10 minutes. Using a standardized WHO nasal spray score checklist. The study found that the intervention on nasal spray usage technique was successful since the overall mean score improved after the intervention (*P* = 0.0001) and the proportion of patients correctly utilizing the nasal spray increased (50.27%). The study found that the inefficient nasal spray application technique was greatly enhanced by the intervention of a pharmacist. In a practical setting, interventional programs improve compliance, and pictorial assistance interventions are more advantageous to patients. A pharmacist-led intervention on nasal spray application technique was found to be beneficial and can greatly increase application accuracy.

Arsoy et al. [40] conducted a cross-sectional study with community pharmacists to evaluate the efficacy of the pharmacist-led educational intervention for enhancing AR patient care. The pharmacists had a thorough, evidence-based 1-day educational training on Allergic Rhinitis and its Impact on Asthma guidelines, and over the course of 6 weeks, they provided patients with a brief education on the management of AR and nasal spray technique. The study found that patients in the intervention group achieved a significantly greater improvement in their QoL and nasal congestion because of the pharmacist-led educational intervention (*P* < 0.05).

## 4. Discussion

Nasal sprays are topical self-medications that are highly effective in treating and increasing the QoL for patients with AR. They release medications to the nasal cavity on a local level, and most of the existing formulations are effective with a single daily dose, demonstrating efficacy, convenience, and safety with few adverse effects [17, 18]. The cornerstones of managing AR still include symptomatic medication, anti-inflammatory therapies, and allergy avoidance. The pharmacological treatment of AR are intranasal steroids and intranasal antihistamines. For the treatment of AR, intranasal corticosteroids administered via nasal spray are the first line of treatment. They are beneficial for treating both mild and moderate-severe AR in both children and adults because they block the invasion of immune cells [41, 42].

Most patients are using nasal spray ineffectively and thereby have poorly managed disease. There are different factors that can be associated with improper application of nasal spray, including patient nonadherence during the prescribed drug period, forgetfulness, inadequate instructions, poor patient-provider interactions, and patients’ disagreement with the need for therapy [23, 24, 33, 35]. Therefore, both patients and healthcare providers should be actively involved in increasing technique teaching. Such patient-motivated and clinician-assisted education could lead to considerable improvements in management as well as wider educational advantages. By ensuring that patients have access to the most up-to-date information, primary care physicians, community pharmacists, and allied health professionals can help reduce the disease burden [43].

From this perspective, it is very important to take medications as directed, as one of the most common mistakes patients make with their medications’ intake is not knowing the benefits of taking their medications, the correct dose, time, frequency, and the planned duration of treatment. In addition, patients who miss or forget to take their medications regularly are less likely to realize the health consequences of medication nonadherence. Furthermore, patients need to be aware of drug adverse effects and know what to do if they occur [44]. In this line, community pharmacists are ideally placed as healthcare professionals able to provide patients with the required information and assistance regarding the correct use of medicines, including topical preparations. Community pharmacists can play an important role in helping patients understand their medications, emphasize how risky it is for patients to share their medicines, and make patients aware of potential adverse drug effects. They can also eliminate this confusion by engaging patients in interactive communications that help improve patient health literacy [29, 45–47].

Moreover, community pharmacists are in a good position to ensure that patients are well-informed about the use of their topical medications before treatment begins, as they are frequently the first healthcare providers patients are reaching [48]. This role is even more critical given that other healthcare providers are regarded as weak in their knowledge of the proper technique and use of topical treatment. Although doctors and nurses are capable to deliver information about topical treatment administration, community pharmacists can play an additional role in repeating the message and ensuring that patients remember and understand what they have been inforemd [49]. It is also worth noting that some topical medications are accessible without a prescription to people who are self-medicating and may not have seen a doctor at all. In these situations, community pharmacists may be the sole healthcare providers who can provide patient counseling on topical medication use in case there is difficulty reaching the defined healthcare professional on time. Pharmacists’ appropriate educational interventions have been found to promote medication adherence. This makes a compelling case for community pharmacists to play a more active role in teaching patients who use topical medications, including nasal spray, especially given that adherence has historically been poor [39, 50].

Health practitioners, including community pharmacists, must continue to assess and reinforce the right technique to improve compliance and proper use. The patient can better grasp the steps of spray application if they have leaflets with a proper demonstration of the technique for using the nasal spray in their local language. The approach should be evaluated again by community pharmacists to improve the right usage technique of the nasal spray and thus increase the treatment effect [24, 50]. Addressing patient misconceptions and minimizing procedures associated with incorrect or underapplied topical preparations is critical to avoiding decreased therapeutic response, treatment failure, and a lower QoL. Meanwhile, community pharmacists should emphasize the need for corrective measures to raise knowledge and awareness of these concerns and encourage patients to invest more time and effort in correcting any available application errors. The need to develop better instructional methods and application techniques through pharmacist-led intervention should also be accompanied by regular assessment and reinforcement of recommendations using verbal and written instructional information. Such measures could be highly effective in significantly improving skills in self-administration, medication adherence, the effectiveness of therapy, and patient outcomes.

## 5. Conclusions

The study highlights that there are many different aspects of application errors encountered by patients while using nasal spray. These include patient nonadherence during the prescribed drug period, forgetfulness, inadequate instructions, poor patient-provider interactions, and patients’ disagreement with the need for therapy. The study also highlights that there is a dearth of publications regarding the involvement of community pharmacists and pharmacist-led interventions for proper technique and application of these topical preparations. In clinical practice, this might indicate the limited role of community pharmacy in recommending correct techniques to ensure patient comprehension, compliance, and satisfaction. Community pharmacists should highlight the importance of corrective actions in raising knowledge and understanding of these issues, as well as encouraging patients to devote more time and effort to addressing any application issues that may exist. The requirement for improved instructional methods and application techniques through pharmacist-led intervention should be complemented by regular assessment and reinforcement of suggestions based on written and verbal instructional information. This should also be accompanied by iterative assessments of patients’ abilities to apply the spray correctly.

## Acknowledgments

The author would like to thank the specialized pharmacist Arueyingho Oritsetimeyin Victoria for her valuable collaboration.

## Conflicts of interest

The author has no financial conflicts of interest.

## Author contribution

Anmar Al-Taie: conceptualization and supervision, writing—collect literature and data, writing—data interpretation, writing—original draft, writing—review and editing.

## Supplementary material

xxxx can be found via https://www.aafp.org/pubs/afp/issues/2000/1215/p2695.html

xxxx

Click here to view

## References

[1] Nielsen-Bohlman L Panzer AM Kinding DA, editors. Health literacy: a prescription to end confusion. Washington (DC): The National Academies Press; 2004. 25009856

[2] Al-Taie A. Reported knowledge and practices towards the proper use of patient information leaflet among university students. Pharm. Educ 2022;22:835-842.

[3] World Health Organization. “The pursuit of responsible use of medicines: sharing and learning from country experiences.” Available from: https://www.who.int/activities/promoting-rational-use-of-medicines. Accessed April, 2023.

[4] Chen J Mullins CD Novak P Thomas SB. Personalized strategies to activate and empower patients in health care and reduce health disparities. Health Educ Behav 2016;43:25-34. 25845376 10.1177/1090198115579415 PMC4681678

[5] Jimmy B Jose J. Patient medication adherence: measures in daily practice. Oman Med J 2011;26:155-159. 22043406 10.5001/omj.2011.38 PMC3191684

[6] Nur Husna SM Tan HT Md Shukri N Mohd Ashari NS Wong KK. Allergic rhinitis: a clinical and pathophysiological overview. Front Med (Lausanne) 2022;9:874114. 35463011 10.3389/fmed.2022.874114 PMC9021509

[7] Licari A Manti S Ciprandi G. What are the effects of rhinitis on patients with asthma? Expert Rev Respir Med 2019;13:503-505. 30947587 10.1080/17476348.2019.1604227

[8] Pawankar R. Allergic diseases and asthma: a global public health concern and a call to action. World Allergy Organ J 2014;7:12. 24940476 10.1186/1939-4551-7-12 PMC4045871

[9] Skoner DP. Allergic rhinitis: definition, epidemiology, pathophysiology, detection, and diagnosis. J Allergy Clin Immunol 2001;108:S2-S8. 11449200 10.1067/mai.2001.115569

[10] Wheatley LM Togias A. Clinical practice. Allergic rhinitis. N Engl J Med 2015;372:456-463. 25629743 10.1056/NEJMcp1412282 PMC4324099

[11] Fröhlich M Pinart M Keller T Reich A Cabieses B Hohmann C Postma DS Bousquet J Antó JM Keil T Roll S. Is there a sex-shift in prevalence of allergic rhinitis and comorbid asthma from childhood to adulthood? A meta-analysis Clin Transl Allergy 2017;7:44. 29225773 10.1186/s13601-017-0176-5 PMC5715620

[12] Pinart M Keller T Reich A Fröhlich M Cabieses B Hohmann C Postma DS Bousquet J Antó JM Keil T. Sex-related allergic rhinitis prevalence switch from childhood to adulthood: a systematic review and meta-analysis. Int Arch Allergy Immunol 2017;172:224-235. 28456795 10.1159/000464324

[13] Li CW Chen DH Zhong JT Lin ZB Peng H Lu HG Yang Y Yin J Li TY. Epidemiological characterization and risk factors of allergic rhinitis in the general population in Guangzhou city in China. PLoS One 2014;9:e114950. 25514026 10.1371/journal.pone.0114950 PMC4267734

[14] Elholm G Linneberg A Husemoen LL Omland O Grønager PM Sigsgaard T Schlünssen V. The Danish urban-rural gradient of allergic sensitization and disease in adults. Clin Exp Allergy 2016;46:103-111. 26096697 10.1111/cea.12583

[15] D’Amato G Akdis C. Global warming, climate change, air pollution and allergies. Allergy 2020;75:2158-2160. 32738058 10.1111/all.14527

[16] Bergmann KC Buters J Karatzas K Tasioulis T Werchan B Werchan M Pfaar O. The development of birch pollen seasons over 30 years in Munich, Germany—an EAACI task force report. Allergy 2020;75:3024-3026. 32575167 10.1111/all.14470

[17] Bridgeman MB. Overcoming barriers to intranasal corticosteroid use in patients with uncontrolled allergic rhinitis. Integr Pharm Res Pract 2017;6:109-119. 29354557 10.2147/IPRP.S129544 PMC5774310

[18] Ehrick JD Shah SA Shaw C Kulkarni VS Coowanitwong I De S Suman JD. Considerations for the development of nasal dosage forms. Sterile Product Develop 2013;6:99-144.

[19] American Family Physician. Nasal sprays: how to use them correctly. Available from: https://www.aafp.org/pubs/afp/issues/2000/1215/p2695.html Accessed April 20, 2023.

[20] Ganesh V Banigo A McMurran AEL Shakeel M Ram B. Does intranasal steroid spray technique affect side effects and compliance? Results of a patient survey J Laryngol Otol 2017;131:991-996. 29050548 10.1017/S0022215117002080

[21] Benninger MS Hadley JA Osguthorpe JD Marple BF Leopold DA Derebery MJ Hannley M. Techniques of intranasal steroid use. Otolaryngol Head Neck Surg 2004;130:5-24. 14726906 10.1016/S0194-5998(03)02085-0

[22] Tay SY Chao SS Mark KT Wang Y. Comparison of the distribution of intranasal steroid spray using different application techniques. Int Forum Allergy Rhinol 2016;6:1204-1210. 27315490 10.1002/alr.21807

[23] Almutairi TA Aldayel AA Aldayel AS Alotaibi F Alhussain HA. Safety concerns of nasal corticosteroids usage in patients with allergic rhinitis. Cureus 2020;12:e11651. 33251078 10.7759/cureus.11651 PMC7686935

[24] Kc B Khan GM Shrestha N. Nasal spray use technique among patients attending the out-patient department of a Tertiary Care Hospital, Gandaki Province, Nepal. Integr Pharm Res Pract 2020;9:155-160. 33062617 10.2147/IPRP.S266191 PMC7519804

[25] Haynes R McDonald H Garg A Montague P. Interventions for helping patients to follow prescriptions for medications. Cochrane Database Syst Rev 2002;2:CD000011. 10.1002/14651858.CD000011 12076376

[26] Ansari M Rao BS Koju R Shakya R. Impact of pharmaceutical intervention on inhalation technique. Kathmandu Univ J Sci Eng Technol 2005;1:1-10.

[27] Rollema C van Roon EN de Vries TW. Inadequate quality of administration of intranasal corticosteroid sprays. J Asthma Allergy 2019;12:91-94. 31040706 10.2147/JAA.S189523 PMC6452790

[28] Maxwell SR. Rational prescribing: the principles of drug selection. Clin Med (Lond) 2016;16:459-464. 27697811 10.7861/clinmedicine.16-5-459 PMC6297291

[29] Al-Taie A Yilmaz ZK. Evaluation of online counselling services based on Turkish web-based pharmacy care setting: a retrospective observational study. Int J Clin Pract 2021;75:e13726. 32956577 10.1111/ijcp.13726

[30] Sanchis J Gich I Pedersen S; Aerosol Drug Management Improvement Team (ADMIT). Systematic review of errors in inhaler use: has patient technique improved over time? Chest 2016;150:394-406. 27060726 10.1016/j.chest.2016.03.041

[31] Mes MA Katzer CB Chan AHY Wileman V Taylor SJC Horne R. Pharmacists and medication adherence in asthma: a systematic review and meta-analysis. Pharmacists and medication adherence in asthma: a systematic review and meta-analysis. Eur Respir J 2018;52:1800485. 29976652 10.1183/13993003.00485-2018

[32] Jia X Zhou S Luo D Zhao X Zhou Y Cui YM. Effect of pharmacist-led interventions on medication adherence and inhalation technique in adult patients with asthma or COPD: a systematic review and meta-analysis. J Clin Pharm Ther 2020;45:904-917. 32107837 10.1111/jcpt.13126

[33] Loh CY Chao SS Chan YH Wang DY. A clinical survey on compliance in the treatment of rhinitis using nasal steroids. Allergy 2004;59:1168-1172. 15461597 10.1111/j.1398-9995.2004.00554.x

[34] Lourenço O Calado S Sá-Sousa A Fonseca J. Evaluation of allergic rhinitis and asthma control in a Portuguese community pharmacy setting. J Manag Care Spec Pharm 2014;20:513-522. 24761823 10.18553/jmcp.2014.20.5.513 PMC10437505

[35] Tan R Cvetkovski B Kritikos V Price D Yan K Smith P Bosnic-Anticevich S. Identifying the hidden burden of allergic rhinitis (AR) in community pharmacy: a global phenomenon. Asthma Res Pract 2017;3:8. 29201385 10.1186/s40733-017-0036-z PMC5696909

[36] Tan R Cvetkovski B Kritikos V Yan K Price D Smith P Bosnic-Anticevich S. Management of allergic rhinitis in the community pharmacy: identifying the reasons behind medication self-selection. Pharm Pract (Granada) 2018;16:1332. 30416632 10.18549/PharmPract.2018.03.1332 PMC6207357

[37] Tan R Cvetkovski B Kritikos V Price D Yan K Smith P Bosnic-Anticevich S. The burden of rhinitis and the impact of medication management within the community pharmacy setting. J Allergy Clin Immunol Pract 2018;6:1717-1725. 29606639 10.1016/j.jaip.2018.01.028

[38] Russo E Giombi F Paoletti G Heffler E Canonica GW Pirola F Mercante G Spriano G Malvezzi L Keber E Sgcp Giua C. Use, abuse, and misuse of nasal medications: real-life survey on community pharmacist’s perceptions. J Pers Med 2023;13:579. 37108966 10.3390/jpm13040579 PMC10142332

[39] Canonica GW Triggiani M Senna G. 360 degree perspective on allergic rhinitis management in Italy: a survey of GPs, pharmacists and patients. Clin Mol Allergy 2015;13:25. 26528081 10.1186/s12948-015-0029-5 PMC4629286

[40] Arsoy G Variş A Saloumi LM Abdi A Abdi A Başgut B. Insights on allergic rhinitis management from a northern Cyprus perspective and evaluation of the impact of pharmacist-led educational intervention on patients’ outcomes. Medicina (Kaunas) 2018;54:83. 30405059 10.3390/medicina54050083 PMC6262628

[41] Sharma K Akre S Chakole S Wanjari MB. Allergic rhinitis and treatment modalities: a review of literature. Cureus 2022;14:e28501. 36185919 10.7759/cureus.28501 PMC9514154

[42] Ramey JT Bailen E Lockey RF. Rhinitis medicamentosa. J Investig Allergol Clin Immunol 2006;16:148-155. 16784007

[43] Al-Taie A. Implications of health care providers by physicians’ and pharmacists’ attitudes and perceptive barriers towards interprofessional collaborative practices. Braz J Pharm Sci 2022;58:1-11.

[44] Brown MT Bussell JK. Medication adherence: WHO cares? Mayo Clin Proc 2011;86:304-314. 21389250 10.4065/mcp.2010.0575 PMC3068890

[45] Rutter P. Role of community pharmacists in patients’ self-care and self-medication. Integr Pharm Res Pract 2015;4:57-65. 29354520 10.2147/IPRP.S70403 PMC5741028

[46] Carlisle A Jacobson KL Di Francesco L Parker RM. Practical strategies to improve communication with patients. P T 2011;36:576-589. 22346326 PMC3278143

[47] Al-Taie A Yilmaz ZK Dahman H Yardimci T. Insights into disease and pharmacotherapy knowledge of Alzheimer’s disease among community pharmacists: a cross-sectional study. Curr Med Res Opin 2022;38:2209-2217. 36189738 10.1080/03007995.2022.2129802

[48] Woodford R Woodford EM Langley CA Marriott JF Wilson KA. Patient knowledge and acceptability of topical corticosteroid preparations: the role of the pharmacist in patient education. Int. J. Pharm. Pract. 2001;9:38.

[49] Tucker R. Community pharmacists’ perceptions of the skin conditions they encounter and how they view their role in dermatological care. Int. J. Pharm. Pract. 2012;20:344-346. 22953774 10.1111/j.2042-7174.2012.00212.x

[50] Lau WM Donyai P. Knowledge, attitude and advice-giving behaviour of community pharmacists regarding topical corticosteroids. Pharmacy (Basel) 2017;5:41. 28970453 10.3390/pharmacy5030041 PMC5622353

